# Reviews

**Published:** 1881-02

**Authors:** 


### Reviews.

---

**A Manual for the Practice of Surgery.** By THOMAS BRYANT, Surgeon to Guy's Hospital, etc. Third American, from the Third Revised and Enlarged English Edition. Edited and Enlarged for the use of the American Student and Practitioner. By JOHN B. ROBERTS, M. D., etc. With Seven Hundred and Thirty five Illustrations. Philadelphia: Henry C. Lea's Son & Co. 1881.

Bryant's Surgery is well known, and highly appreciated by the profession as one of the standard works used or recommended in almost every college as a text book. The third edition has been carefully revised by the author, and many additions, besides numerous new illustrations, added, so that the work represents, in a concise and practical form, our present knowledge in this important branch of medical science. Some parts, we take the liberty to state, might with propriety have been left out, as for instance, the whole subject of ophthalmology, treated in about 70 pages. Every surgery contains the same short treatise on the subject, without going sufficiently into detail to be of any practical value, either for the student or practitioner. For such knowledge, we must necessarily go to the manuals on the subject; a little knowledge is often worse than none at all. Dr. Roberts, the American editor, has introduced many valuable additions, relating especially to the opinions of American surgeons. We predict for the publishers a large sale, as the work richly deserves.

---

**How to use the Forceps**; with an Introductory Account of the Female Pelvis and the Mechanism of Delivery. By HENRY G. LANDIS, A. M., M. D., Professor of Obstetrics and Diseases of Women and Children in Starling Medical College. Illustrated. New York: E. B Treat, Publisher, 757 Broadway. 188[illegible].

The author of this little work of 168 pages presents some practical thoughts upon the mechanism of labor and the use of the Forceps. What object was in view in its preparation for the press, especially when all the principles he endeavors to elucidate are more exhaustively treated in the larger and more comprehensive works is not plain, unless to present in a concise form his own teachings to the students to whom he lectures. If such were the object, we think the work may be of use, especially to the class interested or impressed with the author's method of presenting the important subjects here treated. The work will also be of use to young practitioners and to those unable to procure more extensive treatises.

---

**Lectures on Diseases of the Nervous System, delivered at La Salpetriere.** By J. M. CHARCOT, Professor to the Faculty of Medicine of Paris, &c Translated from the Second Edition By GEORGE SIGERSON, M. D., M. Ch. with illustrations. Philadelphia: Henry C. Lea 1879.

Prof. Charcot treats of diseases of the nervous system in this work, under three heads: first, disorders of nutrition consequent on lesions of the brain and spinal cord; second, paralysis agitans and disseminated sclerosis; third, hysteria and hystero-epilepsy. This classification enables the distinguished lecturer to unravel many of the intricacies of the subject of which he treats, and to give a clearer exposition of the neuroses than we find in any other work. This would be expected of any effort upon the part of one whose reputation has become world-wide. We will not attempt a review of this excellent work. It is such as the profession needs and its timely publication supplies a void long felt in the study and treatment of nervous diseases. It is by all means the most concise and lucid treatise upon these subjects we have had the opportunity to examine.

---

**Diseases of the Pharynx, Larynx and Trachea.** By MORELL MACKENZIE, M. D., London, Senior Physician to the Hospital for Diseases of Throat and Chest, etc. New York: William Wood & Co. 1880.

This work, one of the series of Wood's library of standard authors, is eminently fitted to have a place in every physician's library. It is based on the courses of lectures delivered by the author during twelve years in the London Hospital Medical College, and on his prize essay, "Diseases of the Larynx," but the larger portion of the work has never before been published. The chapters on Diphtheria and Laryngo-tracheal Diphtheria, "formerly called croup," are especially interesting. The author takes the stand, and it seems to us with propriety, that croup is the same disease as diphtheria, and that the supposed pathological and clinical differences are the result of the different anatomical structures, and their connection with the lymphatic glands of the pharynx and larynx. The book is well illustrated, and will occupy a foremost place in the literature of this subject.

---

**Is Consumption Contagious?** By H. C. Clapp, M. D. Boston and Providence: Otis Clapp & Son. 1881.

This little book of 170 pages is a compilation or collection of data tending to show the communicability of consumption. It also contains chapters upon the inoculability of tubercle, and the possibility of transmitting tuberculosis by means of food. Considering the amount of evidence required to establish the most insignificant point in pathology, it can hardly be said that the author positively proves his case, and yet the cases adduced tend strongly in that direction. That consumption is somewhat communicable, is, we believe, the view entertained by the profession generally.

---

**Ophthalmic Test Types.** New York: William Wood & Co.

Many an estimable practitioner might glance at this cumbersome boxfull, and wonder what special use he could make of graduated types and of queer looking diagrams, accompanied with a few lenses and a bunch of variegated yarns. If he knew anything though, he would recognize the types as in most of the text books on ophthalmology, and by some effort of the memory could recall how near and far-sightedness were to be detected and corrected by glasses. There are unfortunately those whose actual knowledge of the errors of refraction and accommodation end here. If they ever tried to read up a suspected case, they soon became discouraged by the prolixity of the larger text books, but especially because they had no suitable lenses or tests at hand. It is to supply just this want that the present set is issued. By means of the glasses and types, the existence, at least, of near and far-sightedness and of astigmatism can be determined, and an approximation can be made as to their degree. This alone is sometimes of decided advantage. In addition, however, there is a set of Holmgrens worsteds for testing color-blindness, a portion of the examination of the eye which is now generally regarded as necessary to a complete knowledge of its condition, and frequently desirable in connection with the employment of the individual. Moreover the box is accompanied by a few pages of elementary suggestions from Professor Noyes as to "How to Choose Glasses," and by an equally concise article by Dr. G. R. Cutter, explaining the use of the "Ophthalmic test types" as a whole. Some improvements might be made by presenting these in a more compact and convenient form, but otherwise they can only be spoken of in terms of commendation.

---

**Minor Surgical Gynecology.** A Manual of Uterine Diagnosis and the Lesser Technicalities of Gynecological Practice. For the use of the Advanced Student and General Practitioner. By PAUL F. MUNDE, M. D., Professor of Gynecology in Dartmouth Medical College, etc., etc. With three hundred illustration. New York: William Wood & Co., 27 Great Jones street. 1880.

This work is one of the "Medical Library," issued by Messrs. Wood & Co. The author is known as the accomplished editor of the *American Obstetrical Journal*, in which the contributions from his pen have been numerous and always of real scientific and professional value and interest. It may be justly expected that the present work would be one of great practical importance, from the well-known reputation of the writer. A careful examination proves such to be the fact. While not as comprehensive in its scope as Emmet's late work, it deals more in the minor details of surgical gynecology, and furnishes to the

"advanced student and general practitioner" the information he is unable to obtain from any other single work, and which is invaluable to him, if removed from opportunities to learn through clinical instruction, the various steps and instruments necessary in operative procedures. We think that in this respect the publishers have been eminently successful in giving to the profession this work, and we have no doubt it will be duly appreciated for the special purpose it aims to supply. The illustrations are numerous, and give valuable assistance in comprehending the operations described, and the instruments required. We recommend the work to the profession.

---

**Medical Heresies: Historically Considered.** A series of critical essays on the origin and evolution of Sectarian Medicine. By GONZALVO C. SMYTHE, A M., M D. Philadelphia; Presley Blakiston.

This book gives, in a small compass, an excellent history of medicine, from its earliest days to the present time, and although of comparatively few pages, contains much that had been elaborated into larger and more pretentious volumes. The author divided his account into three distinct periods, the mythological, the dogmatic and the rational; and it is curious, as well as instructive, to trace the gradual evolution of the science from the nebulous superstitions of the Egyptians, through the slowly-lifting clouds of ignorance of the middle ages, down to the fuller development of to-day. Nor can we flatter ourselves, it has wholly emerged from its whilom hazy condition. In spite of the fanaticism of the Egyptians, medicine surely owes much to them, probably more than the author gives them credit for. He shows clearly, however, how the industry of Galen left its deep impress on the study, although he flourished early in the dogmatic era. The rational age is dated from the closing of the eighteenth and the beginning of the nineteenth centuries, and in it is observable the rapid advancement made, not only in medicine, but in all the collateral sciences. As knowledge lifts the veil and disease is discovered to be not an evidence of the blind displeasure of the gods, but a result of the violation of the God of Nature's immutable laws, so do medical heresies proportionally diminish, and progress is astonishingly accelerated. The latter portion of the book is devoted to a discussion or rather chronicle of the principles of homœopathy. The author endeavors to judge of this school from an unprejudiced standpoint, and the presentation of his concise views will be acceptable to that class, desiring to gain an insight into the mere history of homœopathy, and to those whose limited business forbids wider investigation. Indeed, the whole volume will undoubtedly be interesting to a large part of the reading public. Its data and statistics, it is true, can be found in most encyclopedias, but are here set forth in popular style, and pleasantly available to all.

---

**Cyclopædia of the Practice of Medicine.** Edited by DR. H. VON ZIEMSSEN. Vol. IX Diseases of the Liver and Portal Vein, with the chapter relating to Interstitial Pneumonia. New York: William Wood & Co.

When the enterprising publisher undertook this elaborate work, there were not a few to predict its failure to meet the wants of American practitioners. The manner in which it has been received, speaks well however, for the average physician of this country. The introduction to the present volume, by Ponfick of Rostock, deals with the anatomy and physiology of the liver, while Thierfelder follows with physico-diagnostic observation concerning it. Heller of Kiel has the chapter on its parasites, and Leichtenstern of Tuebingen gives the "clinical aspects of cancer of the liver." The bulk of the work, however, is furnished by von Schueppel of Tuebingen. His chapter on the "pathological anatomy of cancer of the liver" was evidently written with special care, and those on "amyloid degeneration," "fatty infiltration" and "pigmented liver" are also very instructive. He contributes too, the portion relating to "diseases of the biliary passages and portal vein," which part will probably find more eager readers than all the rest of the book. It is fortunate such a work has been placed within the reach of the profession generally, since the liver has from time immemorial been made the scape-goat of many ills flesh is heir to; and a better knowledge of its condition in health and disease ought to relieve it from bearing the burden of other surviving. organs. It may be urged that the writers expend too much time and space on the pathology of the liver, to the exclusion of its treatment under various phases; but we should not forget that a thorough understanding of any disease is absolutely necessary before it can be intelligently dealt with. This volume completes the series of seventeen, and in the circular accompanying it, the publishers show by a few figures how great have been their endeavors to make the work as creditable to themselves, as it is invaluable to physicians.

---

**The Brain as an Organ of Mind.** By H. CHARLTON BASTIAN, M. A., M. D., F. R. S. New York: D. Appleton & Co. $2.00.

Since the announcement was first made, that Bastian was at work at this subject, there has been considerable interest to see the result. His name alone carries much weight with it, standing as he does among the most advanced scientific men of the day. It would have been a disappointment, therefore, to a large circle of readers if the book had been less than a careful digest of the most important facts relating this phase of psychology. For mental phenomena may be viewed from three different aspects. We may study them *objectively*, as manifested in the spontaneous actions of individuals other than ourselves, or in animals; we may examine them *subjectively*, as apparent to our own consciousness, or finally, we may regard them simply as the function of an organ which we call the brain. The two former methods of study have been in vogue for several thousand years, during which time the mind has been looked upon as a mysterious something, which it would be almost heretical, and surely "materialistic" to investigate by ordinary methods. But during the last half century especially, men have begun to regard the brain as simply an organ, whose function we call the mind. It is this relation between anatomy and physiology with which, of course, the writer deals. He is free to admit how little is thus far known in comparison to that yet to be learned, but the present condition of knowledge of the subject is here formulated in a clear and admirable manner. The actual facts contained in such a book could be studied with advantage by classes in mental and moral "philosophy," which are to-day wrestling with impossible problems proposed centuries ago.

---

**Some Limits in the Use of the Ophthalmoscope.** By W. H. Carmalt, M. D.

This is the title of a very excellent article which forms part of the proceedings of the Connecticut Medical Society. In it Professor Carmalt, of New Haven, exposes, in a straightforward way, the impossible pretensions of some writers who assume to recognize various conditions of the brain by an examination of the eye. There is probably nothing that so hinders the advance of any department of medical science as hasty conclusions drawn from insufficient data, and it would seem that in these some of the so-called "cerebroscopists" delight to revel. But unless the state of the retina and optic nerve, in health and disease, is thoroughly understood, it is evidently impossible for the observer to determine whether the changes found in these structures, or in the blood-vessels supplying them, are due to an affection of the eye itself, or of the brain. It is generally conceded that the ophthalmoscope ranks with the use of anæsthetics, and with other great discoveries of this century, as a triumph of modern medicine. But because a new and attractive field has thus been opened to our view, it does not follow that we can look through the eye indefinitely beyond. If so, we might soon hope to peer still further—even through the skull, down along the vertebral canal, and thus perhaps to diagnose affections of the sacral nerves.

